# Interictal blood–brain barrier dysfunction in piriform cortex of people with epilepsy

**DOI:** 10.1002/acn3.52176

**Published:** 2024-08-27

**Authors:** Freya Schulte, Johannes T. Reiter, Tobias Bauer, Julia Taube, Felix Bitzer, Juri‐Alexander Witt, Rory Piper, Anoja Thanabalasingam, Randi von Wrede, Attila Racz, Tobias Baumgartner, Valeri Borger, Louisa Specht‐Riemenschneider, Hartmut Vatter, Elke Hattingen, Ralf Deichmann, Christoph Helmstaedter, Alexander Radbruch, Alon Friedman, Rainer Surges, Theodor Rüber

**Affiliations:** ^1^ Department of Neuroradiology University Hospital Bonn Bonn Germany; ^2^ Department of Epileptology University Hospital Bonn Bonn Germany; ^3^ Developmental Neurosciences UCL Great Ormond Street Institute of Child Health London UK; ^4^ Department of Neurosurgery University Hospital Bonn Bonn Germany; ^5^ Center for Medical Data Usability and Translation Bonn Germany; ^6^ Department of Neuroradiology Clinics of Johann Wolfgang‐Goethe University Frankfurt am Main Germany; ^7^ Brain Imaging Center Goethe‐Universität Frankfurt Frankfurt am Main Germany; ^8^ German Center for Neurodegenerative Diseases Bonn Germany; ^9^ Department of Brain and Cognitive Sciences Ben‐Gurion University of the Negev Beer‐Sheva Israel; ^10^ Department of Medical Neuroscience Dalhousie University Halifax Canada

## Abstract

**Objective:**

The piriform cortex is considered to be highly epileptogenic. Its resection during epilepsy surgery is a predictor for postoperative seizure freedom in temporal lobe epilepsy. Epilepsy is associated with a dysfunction of the blood–brain barrier. We investigated blood–brain barrier dysfunction in the piriform cortex of people with temporal lobe epilepsy using quantitative T1‐relaxometry.

**Methods:**

Gadolinium‐based contrast agent was administered ictally and interictally in 37 individuals before undergoing quantitative T1‐relaxometry. Postictal and interictal images were co‐registered, and subtraction maps were created as biomarkers for peri‐ictal (∆qT1_interictal‐postictal_) and interictal (∆qT1_noncontrast‐interictal_) blood–brain barrier dysfunction. Values were extracted for the piriform cortex, hippocampus, amygdala, and the whole cortex.

**Results:**

In temporal lobe epilepsy (*n* = 14), ∆qT1_noncontrast‐interictal_ was significantly higher in the piriform cortex than in the whole cortex (*p* = 0.02). In extratemporal lobe epilepsy (*n* = 23), ∆qT1_noncontrast‐interictal_ was higher in the hippocampus than in the whole cortex (*p* = 0.05). Across all individuals (*n* = 37), duration of epilepsy was correlated with ∆qT1_noncontrast‐interictal_ (*ß* = 0.001, *p* < 0.001) in all regions, while the association was strongest in the piriform cortex. Impaired verbal memory was associated with ∆qT1_noncontrast‐interictal_ only in the piriform cortex (*p* = 0.04). ∆qT1_interictal‐postictal_ did not show differences in any region.

**Interpretation:**

Interictal blood–brain barrier dysfunction occurs in the piriform cortex in temporal lobe epilepsy. This dysfunction is linked to longer disease duration and worse cognitive deficits, emphasizing the central role of the piriform cortex in the epileptogenic network of temporal lobe epilepsy.

## Introduction

Temporal lobe epilepsy (TLE) is the most common form of drug‐resistant epilepsy in adults. Surgical resection of the hippocampus and amygdala in TLE leads to postoperative seizure freedom in 60–70% of cases.^1^ In addition to the hippocampus and the amygdala, the piriform cortex (PIC) is thought to be highly epileptogenic.^2^, ^3^, ^4^ The PIC is located between the frontal and temporal lobes at the base of the skull and forms the largest part of the olfactory cortex. It has been suggested that the potential epileptogenicity of the PIC may be due to its central location at the anatomical junction between limbic and cortical networks.^5^ Clinically, it has been shown that incomplete resections of the PIC after selective amygdalahippocampectomy or anterior temporal lobectomy are associated with poorer seizure outcome.^3^, ^6^ However, the pathomechanical role of the PIC in human TLE remains unclear.

Previous studies in animal models have demonstrated that early blood–brain barrier dysfunction (BBBD) in the PIC is a predictor of developing epilepsy.^7^, ^8^ Moreover, after drug‐induced generalized seizures, BBBD specifically in the PIC, amygdala, and striatum have been shown.^2^, ^4^, ^9^

In people with epilepsy (PWE), BBBD can be detected in vivo and noninvasively using contrast‐enhanced magnetic resonance imaging (MRI). Physical properties of the tissue can be assessed by directly measuring certain brain tissue parameters such as the T1 relaxation time (quantitative T1; qT1; i.e., T1 relaxometry).^10^, ^11^ Gadolinium causes a notable decrease of the T1 relaxation time. Consequently, extravascular leakage of gadolinium due to BBBD can be detected by a reduction in local T1. This method has already been used to prove that single epileptic seizures are anatomically and temporally associated with BBBD,^12^, ^13^ which appears to persist interictally.^14^ The role of BBBD in epileptogenesis, ictogenesis, and/or related neurological dysfunction of people with epilepsy, however, has not yet been fully elucidated. In this study, we performed T1‐relaxometry (qT1) after both ictal and interictal injection of gadolinium‐based contrast agent in PWE and derived markers of peri‐ictal and interictal enhancement/blood–brain barrier dysfunction in the PIC, the amygdala, the hippocampus, and the whole cortex. We aimed to test the hypothesis that BBBD in the PIC of individuals with TLE occurs peri‐ictally, persists interictally, and is related to disease duration and cognitive deficits in TLE.

## Materials and Methods

### Study group and design

People with epilepsy who underwent presurgical evaluation at the Department of Epileptology at the University Hospital Bonn were prospectively included. Inclusion criteria were (1) age ≥18 years, (2) no contraindications for MRI, (3) no contraindications for gadolinium‐based contrast agent, (4) serum creatinine <1 mg/dL, and (5) absence of mental disabilities. As part of the presurgical evaluation, a multimodal diagnostic assessment including clinical assessment, brain MRI, video EEG monitoring with analysis of semiology, and neuropsychological testing was conducted to identify the presumed seizure onset zone. Subgroups were built according to the clinical focus hypothesis, which was established by a team of physicians with expertise in epilepsy. We extracted this hypothesis from the individuals' health records for our analysis. For the analyses, PWE were organized in subgroups according to the presumed seizure onset zone and clinical information on duration of epilepsy, side of the presumed seizure onset zone and neuropsychological impairment (see Table S1).

All participants underwent a minimum of three MRI sessions: One contrast‐enhanced qT1 scan after both ictal and interictal injection of contrast agent, respectively, was conducted additionally to one precontrast qT1 scan. A standard dose of 7.5 ml of the gadolinium‐based contrast agent Gadovist® (1 mmol/ml Gadobutrol) diluted with 40 ml saline solution was administered to the patient through a peripheral venous catheter. It was followed by 50 ml pure saline solution for flushing. After ictal injection, the patient was examined by a physician and transported to the MRI facility as soon as the clinical condition permitted. All patients were medically supervised by a physician during the transport to the MRI facility and the MRI scan. The interictal injection took place in the MRI facility. Interictal scans were performed at least >24 hours with no clinical seizure (median = 6 days, [range = 1–74]), postictal scans were performed (median 34.6 minutes [range = 18–266]) after seizure onset, nonenhanced qT1 scans were performed >12 hours with no clinical seizure. The data analyzed in this study have partially been analyzed and published by two previous studies, in which interictal contrast‐to‐qT1 latencies were deliberately matched to the postictal contrast‐to‐qT1 latencies.^13^, ^14^ We included the matched latencies in our analyses (see Table 1). Seizure duration, injection latency, and seizure type were assessed retrospectively from the video EEG recordings. The study was approved by the University Hospital Bonn Institutional Review Board. Written informed consent was obtained from all people participating in this study.

**Table 1:** Statistics and clinical characteristics.

Descriptive statistics and clinical characteristics (*n* = 37)	
Female/male	19/18
Mean age at scan	30 [18–51] years
Mean Verbal Memory Score	90 [59–110]
Mean Figural Memory Score	88 [51–110]
Mean duration of epilepsy	15 [2–44] years
Mean seizure duration	00:00:47 [00:00:05–00:03:56] h:min:sec
Mean injection scan latency	
I. Interictal	34.5 [18–266] min
II. Postictal	34.6 [18–266] min
Time intervals between scans	
I. Interictal:noncontrast	1 [0–155] days
II. Interictal:postictal	4 [2–74] days
III. Postictal:noncontrast	3 [0–151] days

± median and range in brackets [].

### Neuropsychological performance

Performance in figural memory was assessed using the revised *Diagnosticum für Cerebralschädigung* (DCR‐R).^15^ Performance in verbal memory was assessed by applying the verbal learning and memory test (VLMT).^16^ In order to correct for age, memory parameters were standardized using a normalization sample of 488 healthy participants (mean = 100, standard deviation = 10). Memory impairment was assumed for scores less than one standard deviation below the mean of the normative sample (<90).^17^

### Quantitative T1 mapping

For each participant, both noncontrast and contrast‐enhanced quantitative T1 maps (qT1) were obtained utilizing a 3 T MAGNETOM Trio MRI scanner (Siemens Healthineers), with a 32‐channel receive head‐coil, following a previously established and optimized protocol.^34^ Quantitative T1 mapping was performed using the variable flip angle method, which involved acquiring of a T1‐weighted gradient echo dataset (TR = 16.4 ms; TE = 6.7 ms; 1 mm isotropic resolution; receiver bandwidth = 222 Hz/pixel; field of‐view = 256 × 224 × 160 mm^3^, acquisition time per flip angle = 4 min 32 sec) while varying the excitation angle. Further information regarding image reconstruction and acquisition parameters can be found in a prior study.^13^

### Image preprocessing

Initial preprocessing was performed using FMRIB Software Library v6.0 (FSL, https://fsl.fmrib.ox.ac.uk/fsl/)^18^ and included brain extraction and linear co‐registration.^19^ Postictal and interictal qT1 volumes were co‐registered to the noncontrast qT1 volumes, and subtraction maps were created (∆qT1_ip_ = interictal – postictal, ∆qT1_ni_ = noncontrast – interictal) in all participants individually. To eliminate the effect of varying central gadolinium concentrations induced by different injection‐to‐scan latencies, pharmacokinetic properties and scan timing between participants, we used the qT1 signal of the superior sagittal sinus (SSS) as internal reference, as it is commonly used for the vascular input function in dynamic contrast‐enhanced MRI.^20^ Therefore, we manually segmented the SSS in three consecutive coronal slices and normalized each ΔqT1 map by the mean intensity of this ROI.

### ROI definition and readout

**Figure 1:**
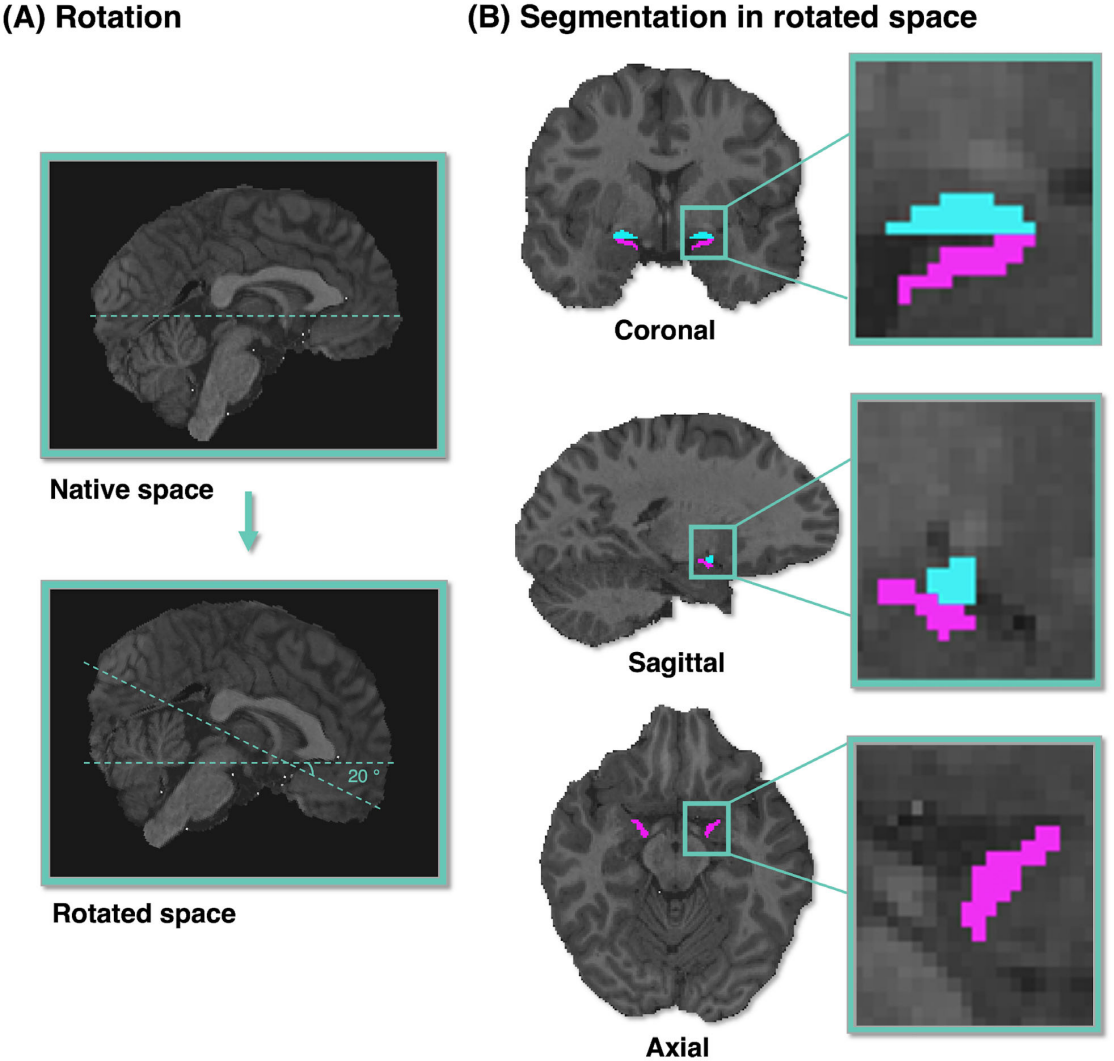
(A) Rotation of the nonenhanced T1‐weighted MRI relative to the anterior commissure by +20°. (B) Sagittal, coronal, and axial view of the piriform masks of a representative subject with epilepsy on a structural T1‐weighted MRI. Color scheme: Blue = frontal PIC; pink = temporal PIC.

Surface reconstruction and tissue segmentation were performed based on the nonenhanced T1 using FreeSurfer (v7.2.0).^21^ From the FreeSurfer‐output, individual masks for hippocampus, amygdala, and the whole cortex were extracted for all participants. The whole cortex is defined as the mask output of the gray cortical matter of FreeSurfer. Amygdala and hippocampus were not included in the mask. The PIC was segmented manually. The PIC was manually outlined on coronal slices of the nonenhanced T1 in a rostral to caudal direction using FSL. The nonenhanced T1 was rotated by +20° relative to the anterior commissure‐posterior commissure axis (fixed point of rotation was the anterior commissure). This re‐angulation facilitates the identification of PIC, amygdala, and hippocampus.^5^ Delineation of the PIC on the rotated nonenhanced T1 was based on previously published methods with anatomical landmarks based on histologic analysis (see Fig. 1).^3^, ^22^, ^23^ Within all ROIs (hippocampus, amygdala, whole cortex, and PIC), the mean gadolinium enhancement (∆qT1_ip_, ∆qT1_ni_) and standard deviation of these regions were readout using *FSL‐stats* and *FSL‐maths*.

### Statistical analysis

Statistical analysis was performed using the Python toolboxes *statsmodels*, *scipy.stats*, *sklearn*, and *scikit_posthocs*. To analyze group differences regarding ROI‐wise ∆qT1_ni_ or ∆qT1_ip_, Kruskal–Wallis tests with Dunn post hoc tests were performed. Effect sizes for group differences are expressed by Glass' ∆, using the whole cortex as reference group. We regard an effect as large if Glass' ∆ >0.8. Linear regression models were applied with ROI‐wise ∆qT1_ni_ or ∆qT1_ip_ as dependent variable. Independent variables were *injection scan latency* and *duration of epilepsy* in all models, and either *presumed seizure onset zone* (TLE versus extratemporal lobe epilepsy = ETLE), *verbal memory* (impaired versus unimpaired), or *figural memory* (impaired versus unimpaired).

## Results

### Clinical characteristics

Thirty‐seven people with drug‐resistant focal epilepsy (19 female, mean age at MRI ± SD: 30.6 ± 8.2 years) were included in this study. 14/37 (38%) participants had TLE and 23/37 (62%) participants ETLE. In 14/37 (38%) of all participants, a seizure onset zone was presumed in the left hemisphere (5/14 of the TLE cohort, 9/14 (64%) of the ETLE cohort). In 14/37 (38%) of all participants, a seizure onset zone was presumed in the right hemisphere (6/14 of the TLE cohort, 8/14 of the ETLE cohort). In 9/37 (24%), no hemisphere could be determined (3/9 of the TLE cohort, 6/9 of the ETLE cohort). In 16/37 (43%) of all participants, the MRI showed no abnormalities (6/16 of the TLE cohort, 10/16 of the ETLE cohort).

### ∆qT1 in TLE and ETLE

**Figure 2:**
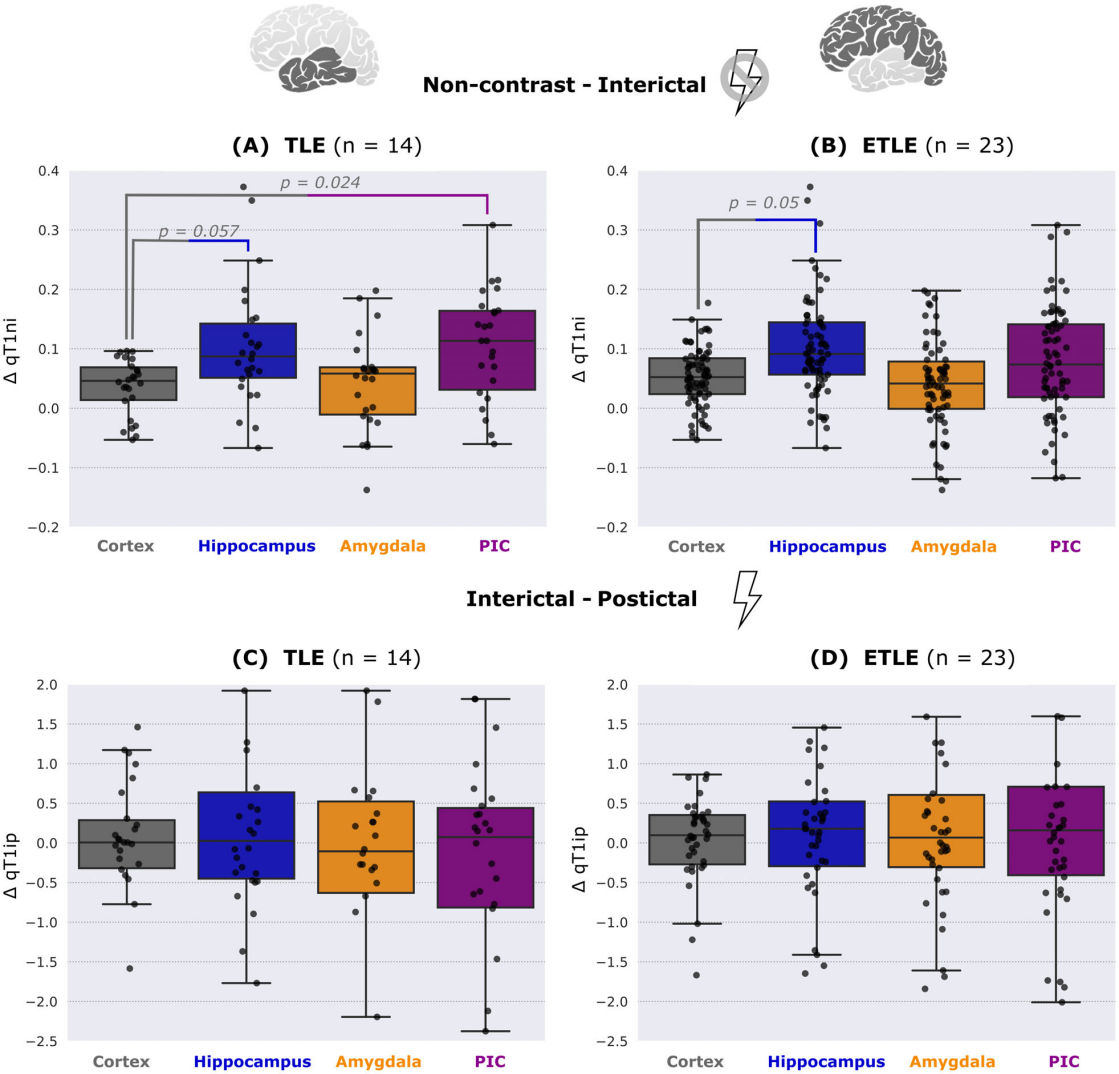
(A) & (B) ∆qT1_ni_ in cortex, hippocampus, amygdala, and PIC of people with TLE and ETLE, respectively. (C) & (D) ∆qT1_ip_ in cortex, hippocampus, amygdala, and PIC of people with TLE and ETLE, respectively. This figure indicates that interictal blood–brain barrier dysfunction occurs in the piriform cortex of people with temporal lobe epilepsy and in the hippocampus of people with temporal and extratemporal lobe epilepsy (please note the borderline‐significant *p*‐values for the hippocampus). Peri‐ictal blood–brain barrier dysfunction is not found. ETLE, extratemporal lobe epilepsy; PIC, piriform cortex; TLE, temporal lobe epilepsy. *p*‐Values <0.1 are shown.

In TLE (*n* = 14), the average ∆qT1_ni_ across both hemispheres was significantly larger in the PIC than in the whole cortex (Dunn post hoc test *p*_PIC_ = 0.024, Glass' ∆_PIC_ = 1.27). Average ∆qT1_ni_ in the amygdala and hippocampus was not significantly different from the whole cortex across both hemispheres but showed a large effect size between hippocampus and the whole cortex (*p*_hippocampus_ = 0.057, Glass' ∆_hippocampus_ = 1.42; *p*_amygdala_ = 1.0, Glass' ∆_amygdala_ = 0.43). In ETLE (*n* = 23), ∆qT1_ni_ was larger in the hippocampus than in the whole cortex (*p*_hippocampus_ = 0.05, Glass' ∆_hippocampus_ = 1.21). ∆qT1_ni_ did not differ significantly between PIC, amygdala, and the whole cortex (*p*_PIC_ = 1.0, Glass' ∆_PIC_ = 0.16; *p*_amygdala_ = 1.0, Glass' ∆_amygdala_ = 0.16). ∆qT1_ip_ did not differ significantly between PIC, amygdala, hippocampus, and the whole cortex neither in TLE nor in ETLE (both Kruskal–Wallis *p* = 1.0, see Fig. 2).

### Association between ∆qT1 and duration of epilepsy

**Figure 3:**
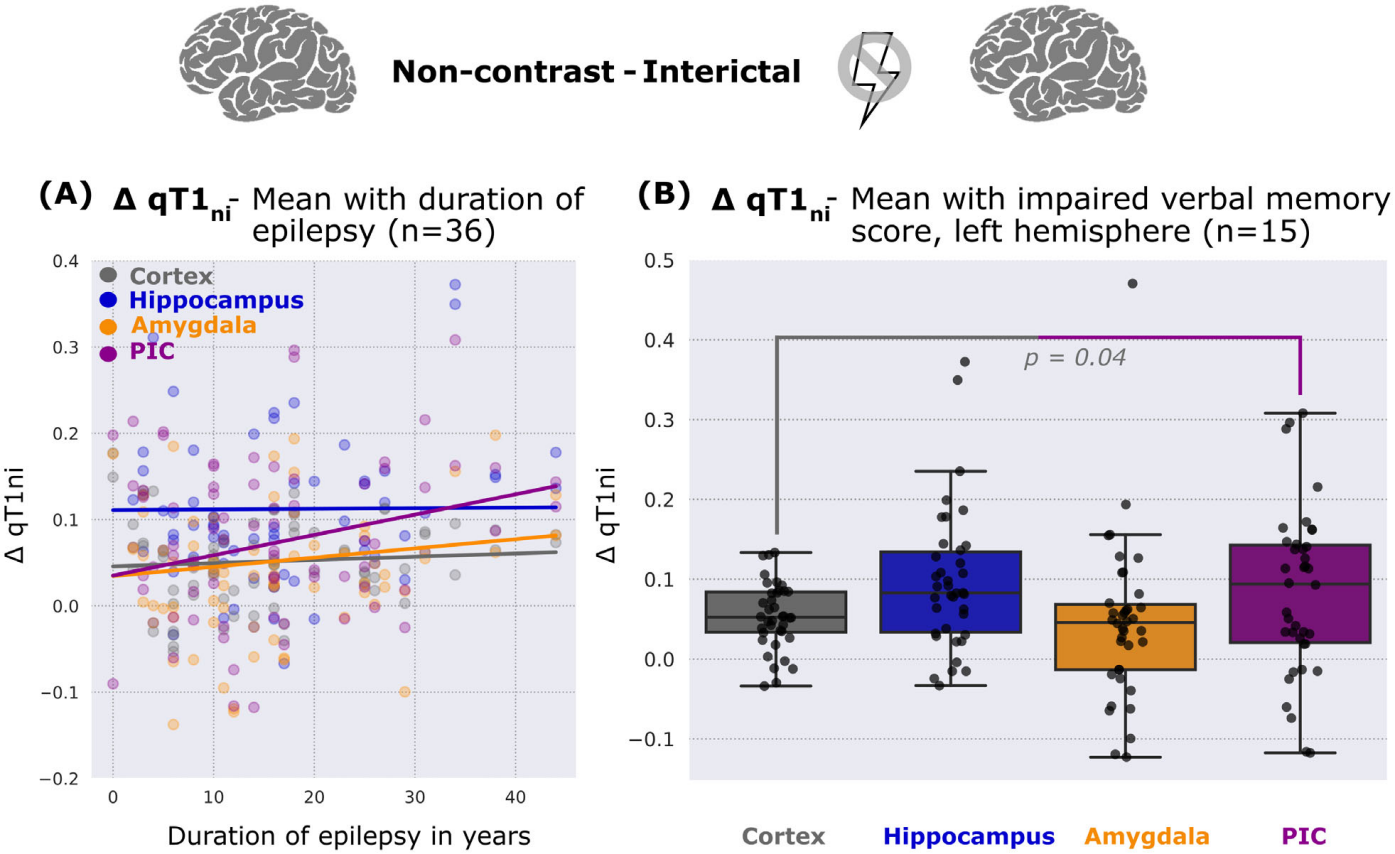
(A) ∆qT1_ni_ in several regions of interest (cortex, hippocampus, amygdala, and PIC) plotted against the duration of epilepsy (in years). (B) ∆qT1_ni_ of people with impaired verbal memory scores in several left hemisphere regions of interest (cortex, hippocampus, amygdala, and PIC). This figure illustrates how duration of epilepsy is correlated with interictal blood–brain barrier in all regions across all individuals, while the association is strongest in the piriform cortex. Also, it demonstrates that people with epilepsy and impaired verbal memory only show interictal blood–brain barrier dysfunction in the piriform cortex. For the purpose of visualization, a smaller scale of the y‐axis in (A) is displayed. Memory impairment was assumed for scores less than one standard deviation below the mean of the normative sample (<90). PIC, piriform cortex. *p* Values <0.1 are shown. The brains above the diagrams indicate the region under investigation.

In the linear regression model across all PWE (*n* = 37) with ∆qT1_ni_ as a dependent variable and duration of epilepsy and ROI as independent variables (“∆qT1_ni_ ~ duration of epilepsy + ROI”), longer duration of epilepsy was significantly associated with a higher ∆qT1_ni_ (*ß* = 0.001, *p* < 0.001) across all ROIs. In a second linear regression model incorporating interaction terms between duration of epilepsy and ROI (“∆qT1_ni_ ~ duration of epilepsy * ROI”), we found no significant difference between regression slopes for PIC, hippocampus, amygdala, and the whole cortex (all *p* > 0.1). However, ∆qT_ni_ in the PIC showed the strongest association with duration of epilepsy having the highest slope (*β*_PIC_ = 0.002, Fig. 3A). In a linear regression model across all PWE with ∆qT1_ip_ as a dependent variable and the interaction between duration of epilepsy and regions of interest as independent variables (“∆qT1_ip_ ~ duration of epilepsy + ROI”), duration of epilepsy was not associated with a higher ∆qT1_ip_ (*β* = −0.023, *p* = 0.5). There was also no significant difference in the slope (“∆qT1_ip_ ~ duration of epilepsy * ROI”) between PIC, hippocampus and amygdala and the whole cortex (*ß*_all rois_ >0.02, *p*_all rois_ = 0.4). In TLE, duration of epilepsy was also associated with a higher ∆qT1_ni_ (*ß* = 0.02, *p* = 0.001), but there was also no difference in the slope (*ß*_all rois_ >0.002, *p*_all rois_ >0.2).

### ∆qT1_ni_ and hemispheres

The hemispheric side of the presumed seizure onset zone was known in 28 individuals. In a linear regression model with ∆qT1_ni_ on the ipsilateral side as a dependent variable and the interaction between injection scan latency and ROI as independent variable, ∆qT1_ni_ in the ipsilateral hemisphere showed significantly greater values in the PIC than in the whole cortex (*ß*_PIC_ = 0.0008, *p*_PIC_ = 0.001), while there was no significant difference between hippocampus, amygdala and the whole cortex (*ß*_hippocampus_ = −0.0005, *p*_hippocampus_ = 0.1; *ß*_amygdala_ = 0.0002, *p*_amygdala_ = 0.416). ∆qT1_ip_ in the ipsilateral hemisphere did not show a significant difference between PIC, hippocampus, amygdala, and the whole cortex (*ß*_all rois_ >0.0007, *p*_all rois_ >0.5).

### ∆qT1_ni_ in PIC is associated with neuropsychological impairment

In participants with impaired verbal memory (*n* = 15, verbal memory <90), ∆qT1_ni_ in left‐hemispheric PIC was significantly higher than in the whole cortex (*p* = 0.04), while it did not show any significant differences between amygdala, hippocampus, and the whole cortex (*p*_amygdala, hippocampus vs. whole cortex_ >0.1; see Fig. 3B). Regarding the figural memory score, we neither found significant differences in ∆qT1_ni_ in the right hemisphere between PIC, hippocampus, amygdala, and the whole cortex in participants with impaired figural memory (*n* = 19, *p* > 0.8), but we found a significant difference in ∆qT1_ni_ in the right hemisphere between hippocampus and the whole cortex (*p*_hippocampus vs. whole cortex_ = 0.02). The comparison of ∆qT1_ip_ in the left hemisphere between the ROIs did not show significant differences in participants with impaired verbal memory (*n* = 15, *p*_all rois_ >0.2). We also did not find differences of ∆qT1_ip_ between ROIs in the right hemisphere in participants with impaired figural memory (*n* = 19, *p*_all rois_ >0.2).

### ∆qT1_ni_ in PIC is associated with neuropsychological impairment in TLE (subgroup analysis)

In a subgroup analysis of people with TLE, ∆qT1_ni_ was significantly greater in the PIC as compared to the whole cortex in people with impaired verbal memory (*n* = 5, *p*_PIC vs. whole cortex_ = 0.003), but did not show significant differences in ∆qT1_ni_ – Mean between hippocampus, amygdala and whole cortex (*p* > 0.3). We did not find differences between ∆qT1_ni_ in the right hemisphere for people with TLE and impaired figural memory (*n* = 6, *p*_all rois_ >0.4). The comparison of ∆qT1_ip_ in the left hemisphere between the ROIs did not show significant differences in people with impaired verbal memory (Verbal Memory impaired and TLE (*n* = 5): *p*_all rois_ >0.3). We neither found differences of ∆qT1_ip_ – Mean on the right hemisphere between ROIs for people with TLE and impaired figural memory (*n* = 6, *p*_all rois_ >0.2).

## Discussion

Previous studies have shown that the PIC plays a key role as a seizure generating and facilitating hub^24^, ^25^ in people with temporal lobe epilepsy.^3^, ^6^, ^26^ A specific dysfunction of the blood–brain barrier in the piriform cortex, however, could only be demonstrated in animal models of TLE.^7^, ^8^ In this study, we hypothesized that peri‐ictal and interictal BBBD is detectable in specific brain regions using quantitative MRI in a cohort of participants with drug‐resistant focal epilepsy.

By comparing the TLE and ETLE subgroups, we found that the PIC in individuals with TLE (but not ETLE) showed a significantly higher ∆qT1_ni_ and thus high contrast agent enhancement than the other ROIs, including the hippocampus, amygdala and whole cortex. This result supports the notion that PIC pathology is specifically linked to TLE.^7^, ^8^, ^27^ Previous work^13^, ^14^ has more generally provided evidence for blood–brain barrier dysfunction in people with epilepsy, which occurs peri‐ictally^13^ and interictally^14^ and co‐localizes with the presumed seizure onset zone. The global analyses applied in previous work, however, fell short of analyzing small anatomical structures of potentially high pathomechanical relevance for epilepsy. The current investigation scrutinizes the PIC, shows specific and functionally pertinent interictal blood–brain barrier dysfunction, thereby emphasizing its pivotal position within the epilepogenic network of temporal lobe epilepsy.

Regarding the duration of epilepsy, we could show that a longer disease duration is associated with a significantly higher ∆qT1_ni_. Even though we could indicate the tendency of PIC showing the strongest association with duration of epilepsy (Fig. 3), we could not find a statistically significant difference in the slope between PIC and whole cortex. This finding can be seen in the context of previous research that stated a correlation between BBBD and exacerbation of epilepsy,^28^, ^29^, ^30^ and a correlation between duration of epilepsy and volume loss or atrophy of mesolimbic structures such as the hippocampus and entorhinal cortex, both of which are connected to the PIC.^23^, ^31^

BBBD has been shown to be associated with cognitive impairment, dementia and Alzheimer's disease.^32^, ^33^ In our study, PWE and with impaired verbal memory showed a significantly higher ∆qT1_ni_ in PIC in comparison with the whole cortex, especially observed in TLE, which is in alignment with the well‐observed notion of verbal memory deficits in cases of left‐temporal pathology.^34^ We did not find any correlation between figural memory and ∆qT1 in our defined ROIs.

We could not detect peri‐ictal BBBD in the defined regions of interest, but interictally. This is consistent with findings in animal studies, where BBBD was detected weeks after an initial epileptic status event.^2^, ^4^, ^9^, ^30^, ^35^, ^36^ Together with the observed association between ∆qT1 values and duration of epilepsy, this allows the interpretation of BBBD as structural sequelae of repeated epileptic seizures.

### Limitations

Manual segmentation of the PIC is difficult because of several factors: First, the PIC is a comparatively small region. The anatomical boundaries of the PIC to other neighboring structures such as the peri‐amygdaloid cortex are difficult to identify at conventional MRI resolutions and there is no standard protocol for manual segmentation, which we based on most recent publications.^22^, ^23^, ^37^

In addition, to capture relatively subtle BBBD, it was necessary to minimize interference of ΔqT1 with any effect other than the one of interest. Otherwise, variations in vascular gadolinium concentrations between participants could mask and obscure BBBD through partial volume effects, particularly those arising from blood vessels. Therefore, normalization of ΔqT1 with a measure of intracranial vascular gadolinium concentration, approximated by the intensity of the SSS, was required. The majority of our analyses were designed intra‐individually and are therefore barely affected by this procedure. However, the normalization of ΔqT1 via the SSS has an impact on the assessment of the association between ΔqT1 and duration of epilepsy and may have caused over‐ or underestimation of ΔqT1.

Since ictal and interictal injection‐to‐scan latencies were largely matched, this resulted in variability of injection‐to‐scan latencies also with respect to interictal analyses (see Table 1). Future studies investigating predominantly interictal qT1 would, therefore, benefit from a protocol that leads to more uniform injection‐to‐scan latencies. Due to the fact that postictal scans are harder to acquire in comparison with interictal or noncontrast scans and may be influenced by peri‐ictal differences in perfusion,^13^ an interictal study protocol with uniform latencies would enhance group comparisons and make analyses more consistent. These studies are needed to validate the findings of this study. Moreover, it should be noted that multiple statistical comparisons conducted in this study increase the likelihood of false positive findings. Finally, we combined different etiologies of TLE in our cohorts. Although this may have increased the variability of the data, it makes our findings generalizable to different types of TLE.

## Conclusions

Our data show interictal BBBD in the PIC and thereby strongly support the conclusion that BBBD in the PIC is associated with chronic epilepsy rather than with individual seizures. The presence of an interictal BBBD in the PIC is specific to the ipsilateral hemisphere and functionally relevant as shown by correlation with cognitive impairment like verbal memory. The detection of a BBBD in the PIC suggests an explanatory contribution to our understanding of the pathophysiology of TLE and adds a clinical diagnostic and therapeutic value. The therapeutic added value is potentially not only exhausted in modified epilepsy surgical protocols, but possibly offers concepts of targeted drug delivery.^38^ Evidence of blood–brain barrier dysfunction in the PIC offers the possibility of an early drug target, which could be both suppression of signal pathways of inflammatory mediators such as TGF‐ß and injection of pharmaceuticals that are not crossing the blood–brain barrier and may, thus, selectively target epilepsy‐affected areas.^39^, ^40^

## Author Contributions

Freya Schulte, Johannes T. Reiter, Tobias Bauer, Rainer Surges, Alon Friedman, and Theodor Rüber contributed to the conception and design of the study. All authors contributed to the acquisition and analysis of data. Text and figures were prepared by Freya Schulte, Johannes T. Reiter, Tobias Bauer, and Theodor Rüber. All authors critically revised the manuscript for intellectual content.

## Conflict of Interest

Juri‐Alexander Witt reports personal fees from Eisai. These activities were not related to the content of this manuscript. Randi von Wrede has received travel support, fees as speaker or for serving on the advisory board from Angelini, Apocare, Arvelle, Cerbomed, Desitin, Eisai, GW pharmaceuticals‐JAZZ pharma, and UCB Pharma. These activities were not related to the content of this manuscript. Valeri Borger has received fees for serving as clinical consultant from Brainlab AG. These activities were not related to the content of this manuscript. Christoph Helmstaedter has received grants from the European Union, travel support from Desitin, honoraria for lectures, counseling, and advisory boards from GW Pharmaceuticals, EISAI, and UCB, as well as license fees from EISAI and UCB. Rainer Surges has received fees as speaker or for serving on the advisory board from Angelini, Arvelle, Bial, Desitin, Eisai, Janssen‐Cilag GmbH, LivaNova, Novartis, Precisis GmbH, UCB Pharma, UnEEG, and Zogenix and grants from the Deutsche Forschungsgemeinschaft (DFG), the Bundesministerium für Bildung und Forschung (BMBF), the Bundesministerium für Gesundheit, and the Marga and Walter Boll Stiftung. These activities were not related to the content of this manuscript. Theodor Rüber declares that the research was conducted in the absence of any commercial or financial relationships that could be construed as a potential conflict of interest. The other authors report no competing interests.

## Supporting information


Table S1.

## Data Availability

The data that support the findings of this study are available on request from the corresponding author. The data are not publicly available as they contain information that could compromise the privacy of research participants.
