# Supplementary material for: Interictal blood–brain barrier dysfunction in piriform cortex of people with epilepsy
Source: Ann Clin Transl Neurol. 2024 Aug 27;11(10):2623–32. doi: 10.1002/acn3.52176 (PMC11514923; doi:10.1002/acn3.52176)
Supplement: Supplementary file 1 — Table S1. [file ACN3-11-2623-s001.docx]

| **Participant** | **TLE or ETLE** | **Duration of epilepsy (years)** | **Sex (male=M, female=F)** | **Age (years)** | **Verbal Memory score** | **Figural Memory Score** | **Seizure-Duration (h:min:sec)** | **Injection-scan-latency ni-ip (min)** | **Injection-scan-latency non-contrast-ni (First scan) in min** | **Structural MRI** | **pSOZ** | **Side** |
| --- | --- | --- | --- | --- | --- | --- | --- | --- | --- | --- | --- | --- |
| 1 | TLE | 31 | F | 34 | 83 | 98 | 00:03:56 | 36 | 36 | non-lesional | temporal | left |
| 2 | TLE | 11 | F | 26 | 93 | 80 | 00:01:01 | unknown | unknown | FCD parahippocampal right | mesiotemporal | right |
| 3 | ETLE | 10 | F | 19 | 103 | 110 | 00:01:00 | 29 | 30 | non-lesional | frontal | Unknown |
| 4 | ETLE | 29 | M | 35 | 87 | 65 | 00:00:36 | 266 | 271 | Heterotopia left-occipital | temporal/occipital | left |
| 5 | ETLE | 11 | F | 20 | 102 | 101 | unknown | unknown | unknown | non-lesional | parietal | unknown |
| 6 | ETLE | 18 | F | 30 | 72 | 76 | 00:00:32 | 31 | 31 | non-lesional | frontal | right |
| 7 | TLE | 2 | M | 27 | 106 | 76 | 00:00:33 | 38 | 45 | non-lesional | mesiotemporal | left |
| 8 | ETLE | 3 | F | 24 | 106 | 104 | 00:00:39 | 29 | 29 | FCD in left medial frontal gyrus | frontal operculum | left |
| 8 | TLE | 17 | F | 31 | 102 | 51 | 00:00:24 | 43 | unknown | FCD in left gyri temporales transversi | temporal | unknown |
| 10 | ETLE | 4 | F | 37 | 94 | 86 | 00:01:18 | 258 | 259 | FCD-suspected lesion left frontal | frontal | left |
| 11 | ETLE | 12 | M | 30 | 76 | 65 | 00:00:47 | 27 | 27 | non-lesional | frontal | unknown |
| 12 | ETLE | 8 | F | 34 | 78 | 92 | 00:00:26 | 32 | 28 | Glioma right sulcus olfactorius | olfactory gyrus/frontal | right |
| 13 | ETLE | 20 | F | 34 | 74 | 75 | 00:01:12 | 37 | 37 | non-lesional | frontal | right |
| 14 | TLE | 16 | F | 40 | unknown | unknown | 00:00:35 | 26 | 26 | Hippocampal sclerosis left | mesiotemporal | left |
| 15 | ETLE | 10 | M | 23 | unknown | unknown | 00:01:14 | 25 | 26 | FCD left frontal | frontal | right |
| 16 | TLE | 14 | M | 39 | 59 | 70 | 00:01:27 | 27 | 30 | Hippocampal sclerosis left | mesiotemporal | left |
| 17 | TLE | 5 | M | 34 | 96 | 99 | 00:00:24 | 25 | 22 | Right temporal lesion (LE) | temporal | right |
| 18 | ETLE | 44 | M | 51 | 88 | 92 | 00:00:24 | 18 | 18 | non-lesional | frontal | left |
| 19 | ETLE | 25 | F | 40 | 104 | 88 | 00:00:47 | 19 | 20 | non-lesional | frontal |  |
| 20 | ETLE | 18 | M | 27 | 88 | 87 | 00:00:40 | 23 | 23 | FCD right frontobasal | frontal | right |
| 21 | TLE | 34 | M | 37 | 63 | 67 | 00:00:05 | 164 | 93 | Hippocampal sclerosis right | mesiotemporal | right |
| 22 | ETLE | 23 | M | 26 | unknown | unknown | 00:01:15 | 26 | 26 | non-lesional | frontal | unknown |
| 23 | TLE | 38 | M | 51 | 98 | 88 | 00:00:36 | 20 | 26 | non-lesional | temporal | unknown |
| 24 | TLE | 6 | M | 27 | 103 | 100 | 00:01:58 | 225 | 225 | FCD right temporo-occipital | temporal/occipital | right |
| 25 | ETLE | 3 | F | 18 | unknown | unknown | 00:00:23 | 20 | 20 | Right parietal lesion | parietal | right |
| 26 | ETLE | 16 | M | 23 | 82 | 55 | 00:01:45 | 46 | 49 | FCD left frontal | frontal | left |
| 27 | ETLE | unknown | F | 30 | 90 | 73 | 00:00:48 | 209 | 213 | FCD left precunues | precuneus | left |
| 28 | ETLE | 6 | M | 20 | 88 | 106 | 00:00:32 | 29 | 14 | FCD cingulate gyrus/frontal | frontal/cingulate | right |
| 29 | ETLE | 14 | M | 33 | 76 | 90 | 00:00:26 | 30 | 30 | non-lesional | frontal | unknown |
| 30 | ETLE | 16 | M | 23 | 81 | 71 | 00:00:34 | 44 | 39 | FCD right frontal medial gyrus | medial frontal gyrus | left |
| 31 | ETLE | 27 | F | 29 | 110 | 106 | 00:01:31 | 64 | 64 | non-lesional | frontal | left |
| 32 | TLE | 10 | F | 22 | 87 | 102 | 00:01:36 | 222 | 218 | non-lesional | temporal | right |
| 33 | ETLE | 25 | M | 27 | 100 | 101 | 00:01:32 | 81 | 81 | Tuberous sclerosis | frontal | unknown |
| 34 | TLE | 6 | F | 38 | 85 | 78 | 00:01:03 | 207 | 227 | non-lesional | mesiotemporal | unknown |
| 35 | TLE | 8 | M | 33 | 100 | 90 | 00:01:25 | 45 | 36 | Left temporal lesion | temporal | left |
| 36 | ETLE | 16 | F | 19 | 98 | 81 | 00:01:47 | 45 | 45 | Right frontal lesion | frontal | right |
| 37 | TLE | 26 | F | 42 | 109 | 103 | 00:02:06 | 66 | 63 | non-lesional | mesiotemporal | right |

**Supplementary Table 1.** **Demographics and Clinical Characteristics** _(_**n=37).** ETLE = extra-temporal lobe epilepsy, FCD = Focal Cortical Dysplasia, LE = Limbic Encephalitis, TLE = temporal lobe epilepsy, pSOZ = presumed seizure onset zone.
